# The Addis Ababa Lions: Whole-Genome Sequencing of a Rare and Precious Population

**DOI:** 10.1093/gbe/evae021

**Published:** 2024-02-01

**Authors:** Marjan Barazandeh, Divya Kriti, Jörns Fickel, Corey Nislow

**Affiliations:** Pharmaceutical Sciences, University of British Columbia, Vancouver, BC, Canada; Department of Biochemistry and Molecular Biology, Faculty of Medicine, University of British Columbia, Vancouver, BC, Canada; Institute for Biochemistry and Biology, University Potsdam, Potsdam, Germany; Department of Evolutionary Genetics, Research Institute for Zoo and Wildlife Research (IZW), Berlin, Germany; Pharmaceutical Sciences, University of British Columbia, Vancouver, BC, Canada; Department of Biochemistry and Molecular Biology, Faculty of Medicine, University of British Columbia, Vancouver, BC, Canada

**Keywords:** African lions, *Panthera leo*, WGS, comparative genomics, exonic nonsynonymous variants

## Abstract

Lions are widely known as charismatic predators that once roamed across the globe, but their populations have been greatly affected by environmental factors and human activities over the last 150 yr. Of particular interest is the Addis Ababa lion population, which has been maintained in captivity at around 20 individuals for over 75 yr, while many wild African lion populations have become extinct. In order to understand the molecular features of this unique population, we conducted a whole-genome sequencing study on 15 Addis Ababa lions and detected 4.5 million distinct genomic variants compared with the reference African lion genome. Using functional annotation, we identified several genes with mutations that potentially impact various traits such as mane color, body size, reproduction, gastrointestinal functions, cardiovascular processes, and sensory perception. These findings offer valuable insights into the genetics of this threatened lion population.

**Significance**

A whole-genome sequencing study on 15 Addis Ababa lions revealed 4.5 million genomic variants, potentially impacting features such as color, size, reproduction, and physiological processes. The study highlights the genetic distinctiveness of this threatened population.

## Introduction

Lions (*Panthera leo*) are one of the world's most iconic and evocative large predators. The fossil record reports evidence of lions dating to 1.5 to 2 million yr ago, with populations that were widespread throughout Europe, Africa, Asia, and North America. Despite their cultural and ecological importance, lion populations have declined dramatically in the past 150 yr, with extinctions and population collapses due to hunting, habitat loss, human–wildlife conflict, and poaching. For example, Asian lions (*Panthera leo persica*) are now limited to a population of fewer than 300 to 400 individuals in India. African lions have also experienced pervasive regional population declines—with 2 tentative subspecies (*Panthera leo leo* and *Panthera leo melanochaita*) having become completely extinct in the wild over the last 150 yr (Barnett et al. 2006; Antunes et al. 2008; Henschel et al. 2014; Armstrong et al. 2020). However, at least 1 such tentative subspecies has survived in a zoo. In 1948, the former Ethiopian Emperor Haile Selassie established a zoo in the Ethiopian capital of Addis Ababa (AA) with 5 male and 2 female lions. This population quickly grew and has remained steady at ∼20 individuals up to the present day, while during the same interval, most wild African lions have become extinct (Tefera 2003). These AA lions, having spent ∼18 generations in captivity, are phenotypically distinct from most other African lions; they are slightly smaller in stature with a lower body mass and, most notably, the males carry distinctive black manes that extend from their shoulders to their stomachs (Tefera 2003; Bruche et al. 2012; Armstrong et al. 2020).

To disentangle if this phenotypic distinctiveness is plastic (i.e. arising from environmental factors) or if it bears an inherited genetic component, genomic DNA sequencing information is required (Anco et al. 2018). Of the comparative genomic studies performed to date, there is evidence of species-specific positive selection in *Panthera* genomes, including genomic adaptations observed in jaguars, leopards, white lions, and snow leopards that may be responsible for physiological adaptations (Cho et al. 2013; Figueiró et al. 2017). A microsatellite and mitochondrial DNA-based analysis already showed that AA lions possess genomic variants that are distinct from other African lions, with private alleles detected at multiple loci (Bruche et al. 2012).

Furthermore, the abovementioned lion population declines could lead to a potentially increased impact of drift and inbreeding. Therefore, deciphering their gene pool has important implications for their conservation management. For example, genetic management strategies such as captive breeding, physical translocation, and assisted reproduction (both within and across populations) can help to maintain (or increase) genetic diversity and reduce the risk of inbreeding and genetic drift commonly observed in small or isolated populations. These strategies, however, rely on a priori knowledge of the genetic diversity and structure of the populations involved, as well as the potential for gene flow between populations (Bertola et al. 2021).

Thus, to explore the genomic features of the distinct AA lion population and unravel the mechanisms driving their adaptive evolution, we performed a whole-genome sequencing (WGS) study. While microsatellites and mitochondrial DNA have provided some information about the structure of populations (Bruche et al. 2012), they are low-resolution markers and thus yield only partial insight into individual and population genetic diversity. Advances in sequencing technologies have enabled the generation of accurate and cost-effective data sets with small amounts of starting material. Accordingly, WGS offers the potential to produce more comprehensive, detailed, and unbiased information about different species, including their evolutionary history, genetic diversity, and potential for adaptive variation.

Here, we present a WGS study on 15 zoo-reared AA lions to investigate their population genetic diversity and to serve as a starting point for a comparative genomic analysis among the African lion, domestic cat, leopard, and panther and different African lion populations or potential subspecies to understand trait evolution. This study contributes to the growing body of research on the genetics of big cats and provides insights into the genetics of a unique and threatened lion population—with the potential to identify targets for conservation management in genetically impoverished populations.

To support ongoing research efforts, we developed an interactive web page containing all the data and links to external data sources. This website can be accessed from http://chemogenomics.pharmacy.ubc.ca/lion-website/.

## Results and Discussion

Since the samples had been collected a few years ago, we ensured that the integrity of the samples remained intact and verified that all 15 individuals belonged to *P. leo*. Species-specific PCR analysis indicated single bands at the predicted size (206 bp) at the African lion–specific mitochondrial gene, *LIHY*, for all samples (supplementary fig. S1A, Supplementary Material online).

Furthermore, to ensure a balanced representation of both sexes, prior to conducting the next-generation sequencing (NGS), we determined the sex of the 15 individuals and found 8 were male (individuals 1, 2, 4, 7, 8, 10, 12, and 14) and 7 were female (3, 5, 6, 9, 11, 13, and 15). All 15 individuals indicated single bands (150 bp) for *KDM5C* as predicted, while only the 8 males generated single bands (417 bp) for the male-specific locus, *DDX3Y* (supplementary fig. S1B, Supplementary Material online).

NGS yielded 30 to 45× coverage for each sample (supplementary table S1, Supplementary Material online), and the high quality of raw reads was confirmed by FastQC v.0.11.9 (Andrews 2010).

### Variant Detection and Comparative Genomic Analysis

The genus *Panthera* comprises 5 big cat species, which have undergone a rapid and recent radiation. Their evolutionary history, however, remains poorly understood (Figueiró et al. 2017). To comprehensively identify unique (and potentially functional) genetic variants in the AA lion population, we performed a comparative genomic analysis by mapping the sequencing reads of 15 AA lions to the African lion (*P. leo*; PanLeo1.0) as well as 3 closely related genomes: leopard (*Panthera pardus*; PanPar1.0), tiger (*Panthera tigris*; PanTig1.0), and domestic cat (*Felis catus*; felCat9). This approach was aimed at capturing species-specific variants that could have phenotypic and/or functional consequences. Furthermore, because the lion reference genome represents the first draft published (Armstrong et al. 2020), comparing it to more established genomes, such as the domestic cat, served to enhance the reliability and robustness of our results. This approach not only ensures the integrity of our findings but also provides a broader genomic context for the AA lion population’s genetic profile, ultimately advancing our understanding of their evolutionary history and potential adaptations.

Additionally, we expanded our data set by including published sequencing data from 6 present-day African lions, comprising 4 wild-born individuals (Tanzania 1 and 2, Botswana 1 and 2) and 2 captive individuals (CAfrica and SAfrica), hereafter referred to as “supplemental lions” (Cho et al. 2013; de Manuel et al. 2020). To allow a side-by-side and comprehensive population comparison, we reanalyzed this data set using our established WGS analysis pipeline.

The African lion reference genome consists of 38 chromosomes, carrying 19,550 coding genes, 2,703 noncoding genes, and a large fraction (42.5%) of repeat elements (Armstrong et al. 2020; Cunningham et al. 2022). We detected variants for each chromosome when mapped to the 4 reference genomes (supplementary fig. S2, Supplementary Material online; Table 1). As expected, the highest number of AA lion variants, as well as the highest number of homozygous variants, was detected when mapped to the domestic cat genome, with numbers becoming lower in the comparisons with the tiger and the leopard genomes. Going forward, we focused on the results derived from aligning with PanLeo1.0, unless specified otherwise.

**Table 1:** Type of different variants in AA lion individuals when aligned to the domestic cat (felCat9), tiger (PanTig1.0), leopard (PanPar1.0), and African lion (PanLeo1.0) reference genomes

	Species			
	Domestic cat (felCat9)	Tiger (PanTig1.0)	Leopard (PanPar1.0)	African lion (PanLeo1.0)
Genome size (kb)	2,521,863	2,391,082	2,578,019	2,406,807
Total variants	39,396,930	21,298,270	14,596,295	4,478,478
Multiallelic sites, %	1.52	3.62	4.51	5.22
Biallelic sites, %	98.47	96.37	95.48	94.77
SNPs, %	89.01	83.63	81.51	77.58
Indels, %	10.78	15.82	17.81	21.81
Mixed (SNPs + indels), %	0.2	0.53	0.66	0.6
Heterozygote variant sites, %	2.77	6.58	9.77	29.21
Homozygote variant sites, %	91.15	77.87	67.8	15.88
Mixed (Het + Hom) sites, %	6.076	15.54	22.42	54.9

**Fig. 1.**
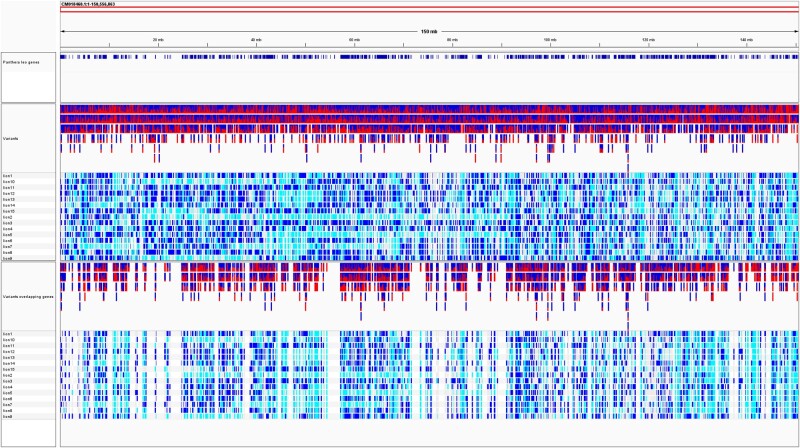
An IGV of the lion chromosome A1, the variants detected for the 15 AA lions, and their overlap with the lion genes (see http://chemogenomics.pharmacy.ubc.ca/lion-website/ for an interactive version of this figure).

**Fig. 2.**
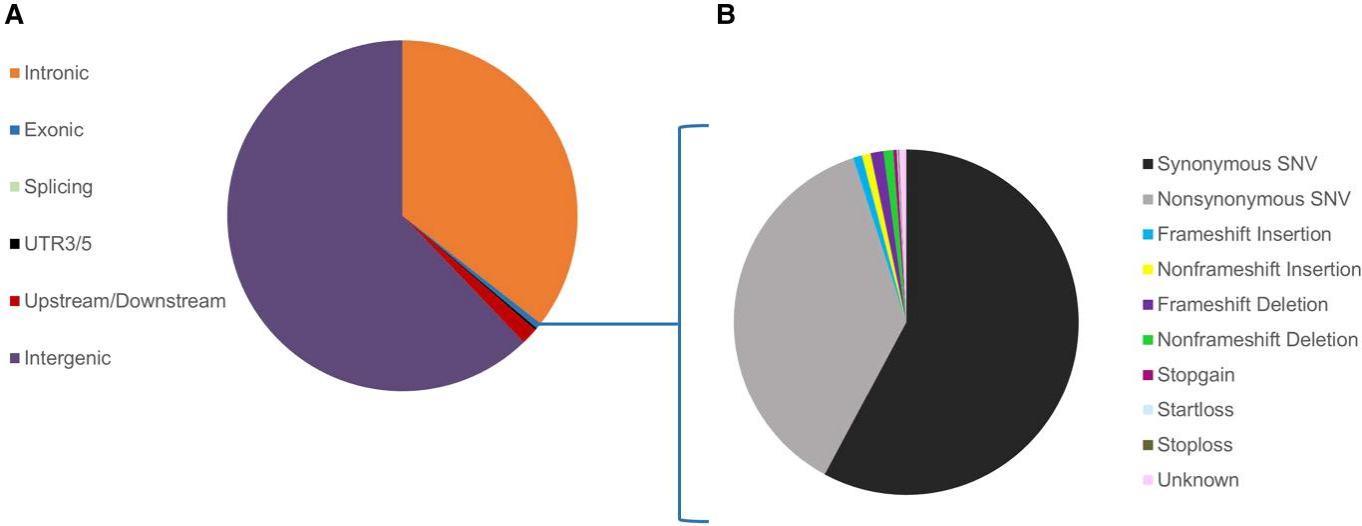
a) Distribution of the variants detected in 15 AA lions across different genomic regions. b) Types of the variants across the exonic regions of the genome.

Among AA lions, we identified 4.5 million variants, with more than 98% of them being called for all 15 individuals, and 27% of them were shared by every individual. These variants were primarily composed of 78% single nucleotide polymorphisms (SNPs) and 22% insertions and deletions (indels). Approximately, 80% of the AA lion genes possessed at least 1 synonymous or nonsynonymous variant (Fig. 1). The majority of variants (62%) are intergenic, with only 0.5% located in exons (Fig. 2a). Among the exonic variants, 58% are synonymous (Fig. 2b).

**Fig. 3.**
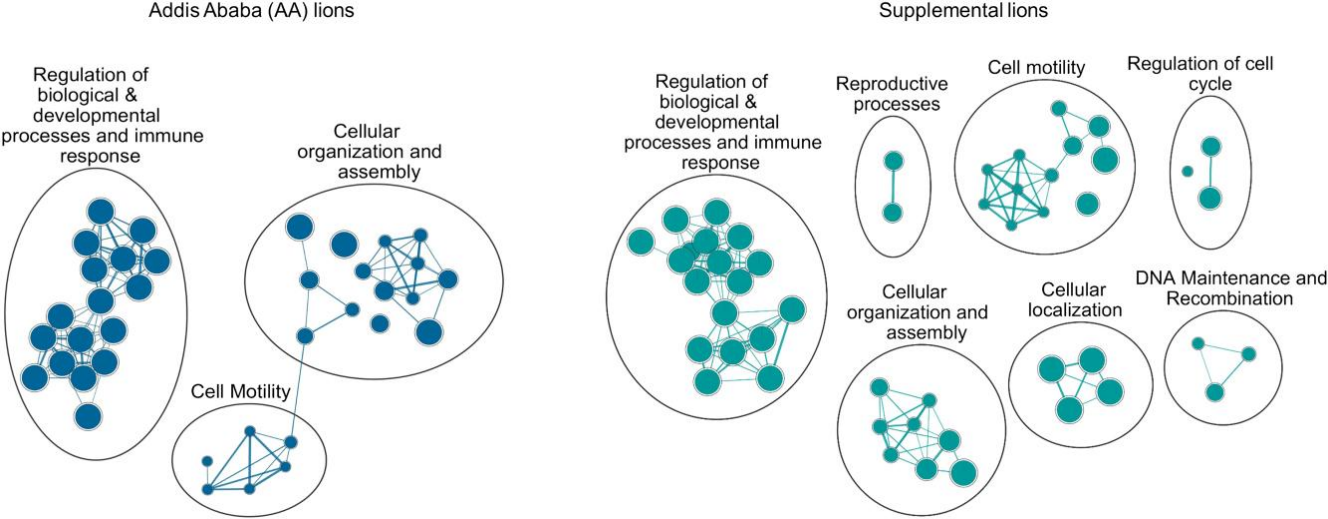
Enriched gene expression pathways based on nonsynonymous SNPs and frameshift indels in AA lions (blue-left panel) and supplemental lions (green-right panel) when mapped to the genome of African lion (PanLeo1.0). The nodes correspond to the number of genes associated with each pathway, and the edges connecting them illustrate the extent of shared genes between the nodes. The width of the edges is proportional to the number of genes that are common between the 2 connected nodes.

When we mapped the genomes of the 6 supplemental lions to PanLeo1.0, we identified a total of 6.5 million variants. Among these, the 2 captive individuals displayed 4.8 million variants, while the 4 wild-born individuals exhibited 5.3 million variants. Of note, AA lions shared 2.9 million variants with the supplemental lions, with 2.5 million and 2.3 million being attributed to the wild and captive individuals, respectively. When we merged these 2 data sets, we isolated approximately 360,000 variants that were exclusive to the captive individuals, encompassing both AA lions and the 2 captives from the supplemental lion group. We then conducted a thorough analysis of all variants, with a specific emphasis on these captive-specific variants to investigate their potential contributions to the traits that may have emerged due to captivity, which will be discussed in more detail below (Fig. 3 and Table 2).

**Table 2:** Genes and variants associated with traits in AA lions and captive supplemental lions

Trait/concern	Gene	Encoding	Chromosome	Position	Mutation	Genotype	Association or role
Mane color	MITF	Melanocyte Inducing Transcription Factor	CM018462.1	33847276	c.G7T:p.A3S	T/T	Pigment deficiency and melanoma
	TYR	Tyrosinase	**CM018470.1**	**41554541**	**c.C176T:p.T59M**	**9(G/A),4(A/A)**	Melanogenesis
Body size	ODAD3	Outer Dynein Arm Docking Complex Subunit 3	CM018462.1	6449959	c.G1156A:p.E386K	4(C/T),11(T/T)	Loss of body fat and body size
Breeding and fertility	ACTL9	Actin-like protein 9	CM018462.1	3773231	c.A344G:p.H115R	3(A/G),12(G/G)	Infertility and sperm defects
			CM018462.1	3773612	c.C725A:p.P242Q	3(C/A),12(A/A)	
			**CM018462.1**	**3773974**	**c.C1087G:p.P363A**	**3(C/G),12(G/G)**	
	ASTL	Astacin Like Metalloendopeptidase	CM018463.1	34800671	c.G1224T:p.Q408H	6(G/T),9(T/T)	
	CFAP54	Cilia and Flagella Associated Protein 54	**CM018467.1**	**115165529**	**c.A6055G:p.K2019E**	**5(A/G),10(G/G)**	
Metabolism	CSF1R	Colony-stimulating factor 1 receptor	**CM018460.1**	**42304012**	**c.C419T:p.P140L**	**6(G/A),9(A/A)**	Diet, glucose metabolism, and lipid metabolic pathways
	ACOT12	Acyl-CoA thioesterase 12	CM018460.1	93753098	c.A365T:p.H122L	5(A/T),10(T/T)	
Cardiovascular	GATA5	GATA transcription factor gene GNC	**CM018463.1**	**135020743**	**c.G783C:p.E261D**	**4(C/G),11(G/G)**	Heart development
	NOTCH1	NOTCH receptor 1	CM018473.1	701639	c.G6898A:p.A2300T	4(C/T),11(T/T)	Cardiovascular morphogenesis
Acoustic responsiveness	CDH23	Cadherin 23	CM018471.1	53296596	c.A4684G:p.I1562V	C/C	Hair cell development and stereocilia formation
Neurological disorders	PEX6	Peroxisome assembly factor 2 protein	**CM018465.1**	**9323733**	**c.A142G:p.T48A**	**C/C**	Retinal degeneration and vision problems
Stress and social behavior	GAD1	Glutamate decarboxylase-67	**CM018468.1**	**61614293**	**c.G1086A:p.M362I**	**5(C/T),10(T/T)**	Anxiety disorders

Genes and variants linked to specific traits in captive lions (AA and captive supplemental lions) are displayed. Genes exclusive to AA lions are highlighted in bold, and any genes with variants found in wild supplemental lions have been omitted.

Genomic variation has been effectively used to pinpoint markers for phenotypic differences in diverse mammals. In dogs, for example, comparative genomic analysis has highlighted markers linked to diverse breed-specific traits (Ostrander et al. 2019; Serres-Armero et al. 2021; Dutrow et al. 2022). Traits like body height and hair length in dogs have been connected to copy number variations (Serres-Armero et al. 2021), or specific genes have been associated with dog fur phenotypes (Cadieu et al. 2009).

Similarly, in big cats, studies have revealed genes responsible for unique adaptations, such as high-altitude survival in snow leopards and coat color in white African lions (Cho et al. 2013; Figueiró et al. 2017). Many other genes contribute to the evolution of distinct feline traits, such as craniofacial development, melanogenesis, and reproduction.

Gene ontology (GO) enrichment analysis of the AA lion and supplemental lion variants identified numerous pathways that are significantly enriched in these populations (Fig. 3). In total, 69 and 108 GO terms were enriched for each data set, respectively (False Discovery Rate < 0.0001; supplementary tables S2 and S3, Supplementary Material online; Fig. 3). Both data sets revealed enriched pathways in developmental processes, signaling and response to stimuli, sensory perception, cell organization and assembly, and cell motility, several of which are potentially connected to reproduction and sperm function, body size, social behavior, and immune response, among others (Wildt et al. 1987; Cho et al. 2013; Gokhale and Shingleton 2015; Maekawa and Inagi 2019; Cook et al. 2022). Supplemental lions also displayed pathways enriched in cell cycle regulation and DNA repair. Upon closer examination of individual variants, we uncovered a subset of variants that were either unique to AA lions or exclusive to captive individuals (AA lions and 2 captive supplemental lions), all of which were associated with these enriched pathways (Table 2).

AA lions possess unique phenotypes, including their distinct mane color and smaller body size compared to other African lions (Bruche et al. 2012). We found mutations in 2 genes—*MITF* and *TYR*—that may be linked to the mane color of AA lions (Jackson 1997; Grill et al. 2013; Figueiró et al. 2017). Further exploration of these genes should provide insights into their role in the evolution of AA lion mane color. Additionally, our analysis identified a gene, *ODAD3*, that is associated with body fat loss and body size (Cunningham et al. 2022).

Because AA lions have been reared in captivity for multiple generations, this environment may induce changes in behavior, phenotype, and molecular characteristics. Indeed, factors such as limited space, the transition from active hunting to regular feeding, and the establishment of daily routines have been identified as contributors to changes in reproductive performance, stress resilience, cardiovascular function, metabolism, and immune response, among others (Clauss et al. 2008; Van der Weyde et al. 2016; Farquharson et al. 2018; Smith et al. 2023). The accumulation of these changes can have detrimental effects on fitness over time. Therefore, studying such mutations in captive animals is crucial for conservation management purposes, because they can provide valuable insights into the effects of captivity on genetic diversity and the long-term health of captive populations (Björklund 2003; Purohit et al. 2021). In our study, we identified specific genetic mutations in genes associated with these traits and other relevant biological processes, which were unique to the captive samples and not present in the wild-born supplemental lions.

Breeding and fecundity can also be affected by captivity. Studies have shown that habitat loss increases the risk of inbreeding, which in turn may impact spermatozoa morphology and function (Wildt et al. 1987; Björklund 2003). As we will discuss in greater detail later, we have found evidence of inbreeding in the AA lions. Furthermore, we observed exclusive variants in the AA lion population and captive supplemental lions’ genetic variants related to sperm motility regulation. These mutations may result in reduced fertility and may have emerged due to captive breeding (Wildt et al. 1987; McKenzie et al. 2015; McKenzie and Lee 2020; Dai et al. 2021; Maddirevula et al. 2022).

Captive animals are also prone to developing gastrointestinal diseases and obesity due to factors such as a sedentary lifestyle and an altered diet compared to that of their wild counterparts (Reeves et al. 2020). Consistent with these observations, we found 2 genes associated with diet, glucose metabolism, and lipid metabolic pathways. Specifically, variants were found in all AA lions, and 1 was also present in the captive supplemental lions (Park et al. 2021; Babaeijandaghi et al. 2022).

Furthermore, chronic stress and a less active lifestyle in captive animals can result in long-term effects on their cardiovascular system. We discovered 2 mutations specific to captive individuals within genes involved in cardiovascular processes, essential for heart development and cardiovascular morphogenesis (Reiter et al. 1999; Ye et al. 2023).

Additionally, signaling and sensory perception were among the enriched pathways in the mutated genes identified in the AA and supplemental lions. Our analysis revealed 1 allelic variant among captive individuals in *CDH23* associated with impaired acoustic responsiveness and hearing loss in other species (Mizutari et al. 2015).

Our study also revealed the presence of 1 AA population–specific nonsynonymous SNP in *PEX6* that has been flagged as associated with neurological disorders, such as retinal degeneration and vision problems (Benson et al. 2021).

Finally, we identified a mutation among AA lions in a gene linked to stress resilience and social behavior, both of which may be influenced by captivity (Donner et al. 2012; Barabas et al. 2021).

Table 2, supplementary fig. S3 and tables S2 to S7, Supplementary Material online, and the anibesa webpage (http://chemogenomics.pharmacy.ubc.ca/lion-website/) provide more details about the mutations mentioned above and other genes of interest as well as enriched GO terms when mapped to different reference genomes.

### Genetic Relatedness and Inbreeding Analysis

To explore the influence of shared ancestry on observed variants in the AA lion population, we calculated the proportion of identity by descent (PI_HAT) among the AA population and when merged with the supplemental lions. No significant relatedness was found within the supplemental lions or between the AA and supplemental lion data sets as all PI_HAT values were 0.

**Fig. 4.**
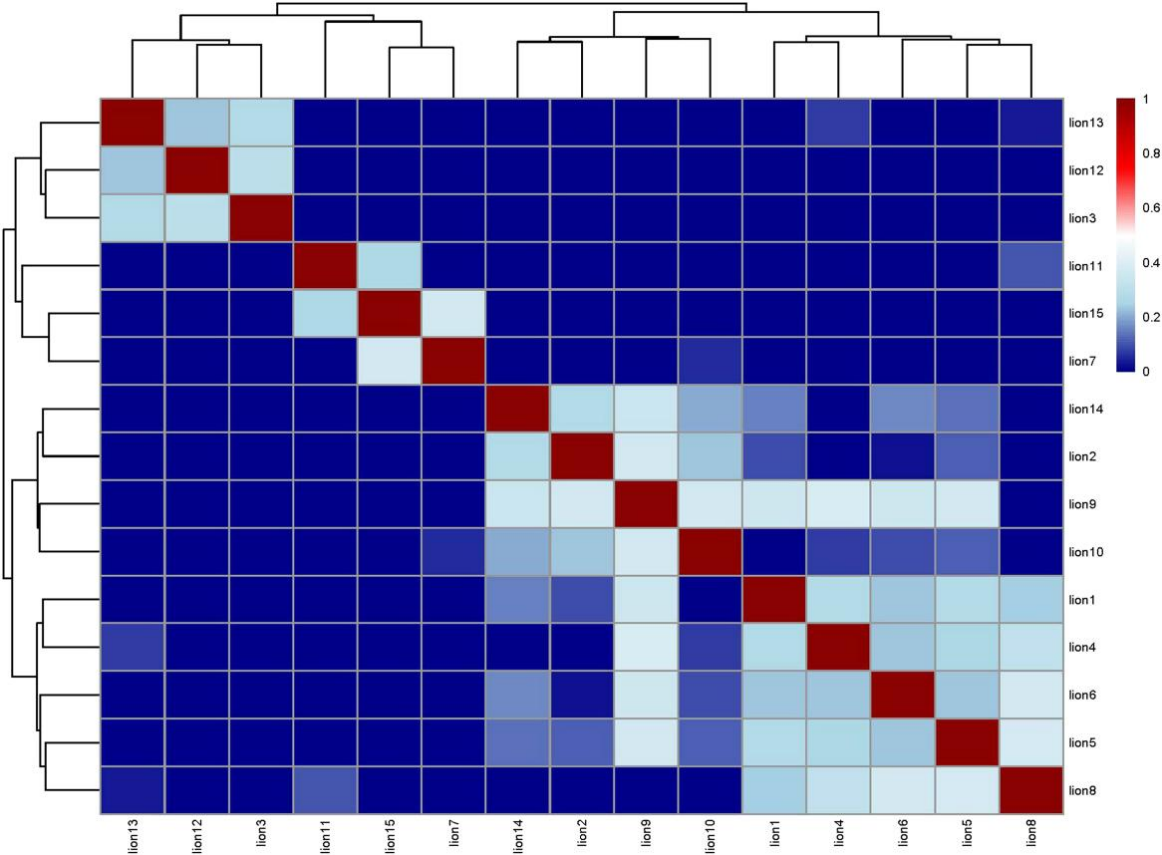
Pairwise relatedness among the 15 AA lions. The heatmap represents the pairwise relatedness values (PI_HAT) between individuals in the AA lion population. The color scale ranges from blue (low relatedness) to red (high relatedness), with white indicating intermediate values. The dendrograms on the top and left sides of the heatmap display the hierarchical clustering of individuals based on their relatedness patterns.

Within the AA lion population itself, we observed intriguing patterns of relatedness. Seventeen pairs (16% of the total) indicated first- and second-degree relatedness with PI_HAT values above 0.25. An additional 21 pairs (20% of the total) showed relationships lower than second degree. Most notably, the majority of the pairs, totaling 67 pairs (64%), appeared to be unrelated to each other (Fig. 4). While this finding suggests that the genetic variants identified in these lions could be partly influenced by familial relationships, our limited evidence of strong relatedness among most of the individuals strengthens the validity of the observed genetic variations, suggesting that the genetic differences observed between the AA lions and other African lions are not necessarily a result of shared ancestry or familial ties. Consistent with this, although there are more than 1.6 million variants specific to the AA lions, several of the variants associated with specific traits are shared between AA lions and captive supplemental lions, despite their lack of relatedness.

When considering restricted populations, it is imperative to consider the possible consequences of inbreeding within the AA lion population. Inbreeding can be considered as mating between genetically related individuals that leads to an increased number of homozygous loci in individuals in a population. To measure the consequences of a reduction in average heterozygosity, we used the “inbreeding coefficient,” which provides the probability of 2 alleles present at the same location being identical in state and descent (Rousset 2002).

**Fig. 5.**
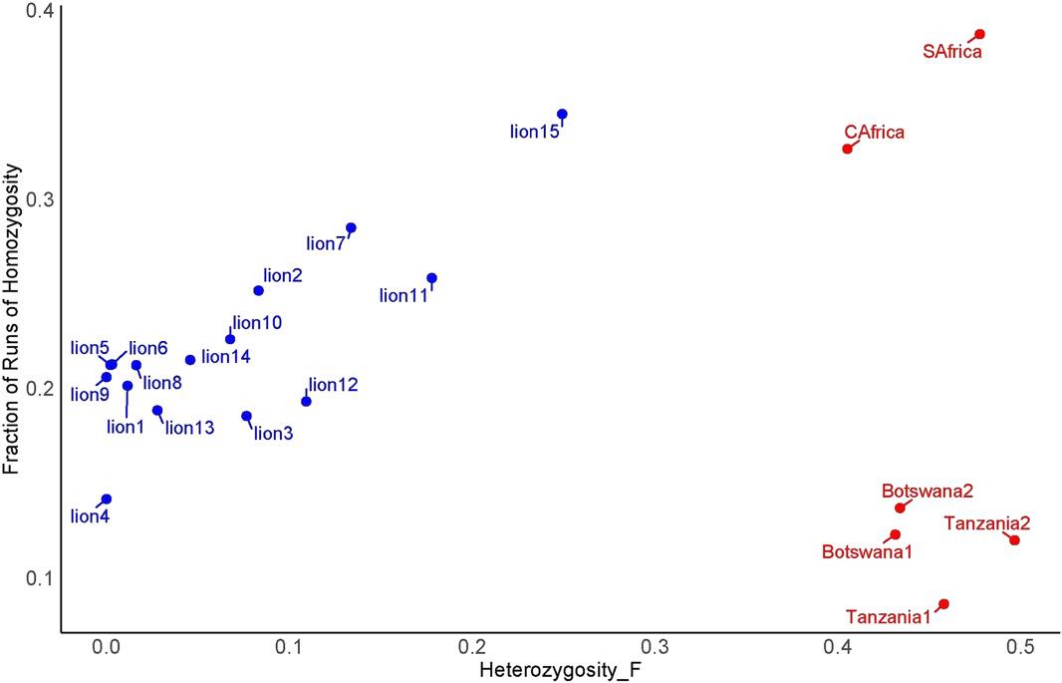
Calculation of inbreeding levels in AA lion as well as supplemental lion populations. The scatterplot illustrates inbreeding measurements based on heterozygosity (Het_F) and *F*_ROH_. The blue dots (Het_F < 0.3) represent individuals from the AA lion population, while the red dots (Het_F > 0.4) represent individuals from supplemental lion populations.

Mammals that have spent decades (and thus numerous generations) in captivity are expected to be inbred to a certain extent (Björklund 2003). In light of this, it is somewhat surprising that a recent study on captive African lions revealed no major signatures of inbreeding compared to the population of wild lions (Miller et al. 2023). Additionally, an earlier study on the AA lions similarly reported an absence of significant inbreeding within this group (Bruche et al. 2012). To reevaluate the extent of inbreeding within the AA lion population, we employed 2 distinct methods: the heterozygosity-based inbreeding coefficient and the fraction of runs of homozygosity (*F*_ROH_). For comparison, we also calculated the same measurements for the supplemental lions and our results were consistent with previously published data (Cho et al. 2013; de Manuel et al. 2020). The heterozygosity-based inbreeding coefficient revealed that, for most of the AA population, inbreeding levels were less than 0.1, and notably smaller than those observed in all supplemental lions, including the wild-born individuals. However, the analysis of *F*_ROH_ indicated a high degree of inbreeding in the majority of AA lions (Fig. 5). All AA lions, except 1, displayed lower inbreeding levels than the 2 captive supplemental lions, and 4 of the AA lions exhibited *F*_ROH_ levels below 0.2, a range similar to that observed in the 4 wild-born supplemental lions. Additionally, only a minor fraction of loci in the AA lion population (1.8%) deviated from the Hardy–Weinberg equilibrium, potentially suggesting random mating among individuals.

As a further validation of our data analysis pipeline and to eliminate potential bias, we applied our pipeline to data from a known inbred puma population from North and South America (Saremi et al. 2019). This analysis also yielded inbreeding levels consistent with their published data, ensuring the reliability of our method (data not presented here).

The discrepancy between inbreeding estimates derived from the 2 methods can be related to the differences in their methodological approaches. The heterozygosity-based inbreeding is calculated based on the overall excess of homozygosity across the genome, without distinguishing between long stretches of homozygosity due to inbreeding and homozygosity resulting from random genetic drift or population structure, potentially leading to underestimation of inbreeding. In contrast, *F*_ROH_ provides a more direct estimate of autozygosity, indicating more recent common ancestry and thus more recent inbreeding. The lengths and distribution of these ROH are less likely to be influenced by random homozygosity or population-specific allelic architecture, making this method potentially more sensitive and accurate for detecting inbreeding within a population, especially with respect to recent inbreeding events (McQuillan et al. 2008; Purfield et al. 2012).

The variance in inbreeding estimates obtained through different methods, as compared to the previous studies (Bruche et al. 2012), underscores the importance of employing a variety of analytical approaches to gain a robust understanding of inbreeding. Our study's use of both heterozygosity-based metrics and *F*_ROH_ analysis illustrates that nuanced insights can be derived from integrating multiple genetic assessment tools.

### Lion Genome Website

Accessing and analyzing genomic sequences and functional data of lions have not been easy for geneticists. We thus have developed the web platform anibesa that provides comprehensive genetic background and generational pedigree information on Ethiopian lions bred at the AA Zoo, enabling users to easily access all our data, query and search remote resources, and visualize the results.

The web portal combines lion genome annotations with a variety of variant analyses to extend the molecular genetic characterization of these lions while making available a user-friendly interactive Integrative Genomics Viewer (IGV; Robinson et al. 2023) instance, with which users can zoom in and out of genomic regions, navigate across chromosomes, and compare different variant data tracks. It also provides links to download reference genome data for the lions and contains GO results showing functional annotation of the genes and related biological processes, molecular functions, and cellular components. anibesa can be accessed at http://chemogenomics.pharmacy.ubc.ca/lion-website.

## Conclusion

Our study provides insights into the genetic makeup of the AA lion population and identifies functional genetic variants (alleles) potentially responsible for unique phenotypic traits. We observed a high number of variants specific to AA lions compared with the African lion and other closely related big cat species, indicating a distinct evolutionary history for this population. Our analysis also identified potential associations between certain genes/alleles and mane color, body size and weight, cardiovascular functions, reproduction, social behavior, stress resilience, diet, and sensory perception, including vision and acoustic responsiveness. Future research will study the biological function of these alleles and their role in the adaptive evolution of this lion population.

## Materials and Methods

### Sample Preparation

Genomic DNA samples were collected as described before (Bruche et al. 2012). Briefly, blood samples from 15 lions were taken and preserved in long-term storage buffer, stored at −20 °C, and transported to the lab, where the DNA was extracted using the DNeasy Blood and Tissue kit (Qiagen). DNA samples were stored at −80 °C until DNA library preparation.

### Genome Integrity and Sex Verification

The integrity of the genomes was confirmed by the amplification of a short fragment (206 bp) of the mitochondrial cytochrome b gene (*LIHY*). The forward (5′-ATGACCAACATTCGAAAATCWC-3′) and reverse (5′-ATGTGGGTSACTGATGAG-3′) primers were designed to be species specific and to avoid amplification of non-African lion species as described (Tende et al. 2014). The 25 μL PCR reaction contained 12.5 μL KAPA HiFi HotStart ReadyMix PCR Kit, 0.3 μM forward and reverse primers, and 50 to 100 ng of the templates. A touchdown PCR was performed with the following cycling reactions: initial denature of 95 °C for 3 min, 20 cycles of 98 °C for 20 s, touchdown annealing of 60 to 50 °C (with a decrease of −0.5 °C/cycle) for 15 s, and 72 °C for 15 s followed by another 20 cycles of 98 °C for 20 s, 55 °C for 15 s, and 72 °C for 15 s with a final extension of 72 °C for 1 min. The amplified fragments were visualized on a 1% agarose gel with SYBR Safe DNA gel staining.

The sex of the 15 AA lions was determined using 2 pairs of primers: *KDM5C* (5′-TGCAAGTGCTCCAGTAGCCG-3′ and 5′-GCAGGGAGCTCATCCAAGGT-3′) and *DDX3Y* (5′-GGTCCAGGAGARGCTTTGAA-3′ and 5′-CAGCCAATTCTCTTGTTGGG-3′) as described before (Tende et al. 2014). The PCR reactions were prepared as described above. A touchdown PCR was performed with the following cycling reactions: initial denature of 95 °C for 3 min, 20 cycles of 98 °C for 20 s, touchdown annealing of 65 to 55 °C (with a decrease of −0.5 °C/cycle) for 15 s, and 72 °C for 15 s followed by another 20 cycles of 98 °C for 20 s, 60 °C (*KDM5C*) or 65 °C (*DDX3Y*) for 15 s, and 72 °C for 15 s with a final extension of 72 °C for 1 min. The amplified fragments were visualized on a 1% agarose gel with SYBR Safe DNA gel staining.

### WGS Library Preparation and Sequencing

Because of the preciousness of these samples, we selected the DNBSEQ PCR-Free method, specifically designed for preparing WGS libraries without PCR amplification (a.k.a. “PCR-free”) for MGI Sequencing Platforms. This method generates DNA nanoballs, which are then sequenced without further amplification, eliminating the effect of clonal errors generated by traditional PCR-based sequencing platforms.

WGS libraries were constructed using MGIEasy FS PCR-free DNA Library Prep Set for all 15 AA lion DNA samples with the goal of achieving 30× coverage for each sample. A total of 1,000 ng of high-quality genomic DNA (OD_260_/OD_280_ = 1.8∼2.0) from each sample was fragmented to obtain a DNA smear that migrated between 150 and 1,000 bp with a peak size between 300 and 500 bp. MGIEasy DNA Clean Beads were used to select a peak size of 475 bp from the fragmented DNA, and the correct selected size was confirmed on an Agilent 2100 Bioanalyzer. End repair, A-tailing, and adapter ligation were performed on 120 to 200 ng of the size-selected samples. Then, the samples were denatured followed by single-strand circularization and Exo digestion. The final products were quantified using Qubit ssDNA Assay Kit, and 75 fmol (12.6 ng of 457 bp DNA) of each library was sequenced on an MGISEQ-2000 PE150 sequencing platform.

### Variant Calling

All reads passed the FastQC tools’ (v.0.11.9; Andrews 2010) quality control check. The reads were aligned to 4 different reference genomes (lion: PanLeo1.0, accession number GCA_008795835.1; domestic cat: felCat9, accession number GCA_000181335.4; leopard: PanPar1.0, accession number GCA_001857705.1; and tiger: PanTig1.0, accession number GCA_000464555.1) using Bowtie2 v.2.3.5 (Langmead and Salzberg 2012). The aligned reads were sorted, and duplicates were marked using Samtools v.1.9 (Li et al. 2009). Picard tools v.2.21.4 (http://broadinstitute.github.io/picard/) were used to add read groups to the bam files.

Variant calling was performed using GATK Best Practices (Van der Auwera and O’Connor 2020). The initial set of variants was called using GATK HaplotypeCaller on GVCF mode for all 15 AA individuals using the 4 reference genomes. Then the individual variant sets were combined and joint genotyped using GATK GenotypeGVCFs for each genome. This single set of variants was used to perform Base Quality Score Recalibration (BQSR) on the uncalibrated bam files. The variant calling and bam recalibration were performed twice until convergence was achieved (supplementary fig. S4, Supplementary Material online). Then the final high-confidence set of variants was generated and filtered using GATK HaplotypeCaller for all 4 reference genomes. These filters were used for the cat, leopard, and tiger genomes: QD (Quality by Depth) < 2.0, MQ (Root Mean Square Mapping Quality) < 30.0, FS (Fisher Strand) > 60.0, SOR (Strands Odd Ratio) > 3.0, MQRankSum (Mapping Quality Rank Sum test) < −12.5, ReadPosRankSum (Read Position Rank Sum test) < −8.0, and Inbreeding Coefficient < −0.3; and these against the lion genome: QD (Quality by Depth) < 2.0, MQ (Root Mean Square Mapping Quality) < 40.0, FS (Fisher Strand) > 60.0, SOR (Strands Odd Ratio) > 3.0, MQRankSum (Mapping Quality Rank Sum test) < −12.5, ReadPosRankSum (Read Position Rank Sum test) < −8.0, and Inbreeding Coefficient < −0.3 (McKenna et al. 2010; DePristo et al. 2011; Van der Auwera et al. 2013; Poplin et al. 2018; Van der Auwera and O’Connor 2020).

The sequencing reads for the supplemental lions (accession numbers SRR11286167, SRR11286168, SRR11286181, SRR11286182, SRR836361, and SRR836370) and pumas (accession numbers SRR7639695, SRR7639696, SRR7542886, SRR7542887, SRR7542888, SRR7660678, SRR7660679, SRR7664677, SRR7664678, SRR7956993, SRR7956994, SRR7610940, SRR7610941, SRR7661934, SRR7661935, SRR7690239, SRR7690240, SRR7543017, SRR7543018, SRR7537344, and SRR7537345) were retrieved from GenBank and aligned to the *P. leo* (PanLeo1.0) and *Puma concolor* (PumCon1.0) genomes, respectively. Subsequently, they underwent the same analysis steps as the AA lions to detect genetic variants.

### Gene Annotation and Trait Assessment

Gtf files of all 4 species were retrieved from the Ensembl database (Cunningham et al. 2022) and used to functionally annotate the obtained variants using ANNOVAR, table_annovar.pl program (version 2020-06-08; Wang et al. 2010). The exonic variants were filtered by retaining only nonsynonymous SNPs and frameshift indels. Functional GO enrichment analysis was performed using g:profiler, v. Ensembl 110 (Bonferroni correction threshold of 0.0001). The BP (biological process) data set was visualized in Cytoscape v.3.9.1 (Shannon et al. 2003) and EnrichmentMap app (node cutoff *q*-value: 0.0001; edge cutoff: 0.25; Merico et al. 2010) and clustered using AutoAnnotate app (Kucera et al. 2016). The variants were filtered further to exclusively contain those present in all 15 AA individuals, aiming to identify genes that include functional mutations contributing to important cellular pathways and distinctive phenotypes of the AA lions. To identify mutations potentially linked to traits specific to captivity, we utilized the “bcftools isec” tool (Danecek and McCarthy 2017) to identify common variants between AA lions and supplemental lions. We then concentrated our analysis on variants present in captive individuals, including both AA lions and captive supplemental lions, for trait assessment.

### Relatedness and Inbreeding

To quantify pairwise relatedness and inbreeding coefficient among AA and supplemental lions, we used PLINK v1.90b6.21 (Purcell et al. 2007).

To determine pairwise relatedness, PLINK calculates identity-by-descent (IBD) estimates. We first performed linkage disequilibrium (LD) pruning with the parameters “--indep-pairwise 50 5 0.2,” which helps to prevent overestimation of IBD statistics resulting from the nonindependent segregation of alleles. Following this, the plink --genome command was used to calculate PI_HAT values, representing the estimated proportion of the genome shared IBD between individual pairs, calculated as (IBD = 2) + 0.5 × P(IBD = 1). To illustrate the relatedness among individuals, we generated a heatmap using the “pheatmap” package in R (version 4.2.1).

To evaluate inbreeding, we employed 2 distinct methodologies in PLINK. First, we used the plink --het command to calculate the heterozygosity-based inbreeding coefficient (*F*) for each individual. This measure is derived from the observed versus expected homozygosity across all loci, providing an estimate of homozygosity that reflects potential inbreeding. An excess of homozygosity, indicated by a positive *F*-value, suggests inbreeding.

We then applied the plink --homozyg command with specific parameters (--homozyg-window-snp 50 --homozyg-window-het 2 --homozyg-window-missing 10 --homozyg-density 50 --homozyg-gap 100 --homozyg-snp 100 --homozyg-kb 1000 --homozyg-window-threshold 0.02 --homozyg-het 750) to identify ROH within the genome. ROHs are uninterrupted sequences of homozygous genotypes that indicate a common ancestry for the alleles in those regions, often resulting from mating between close relatives. Then the fraction of the genome in ROH (*F*_ROH_) was calculated, which provides a direct genomic measure of inbreeding. We then created a scatterplot in R (version 4.2.1) to display the relationship between the heterozygosity-based *F* and *F*_ROH_ using the package “ggplot2.”

### Website Development

The layout for the “anibesa” website and its user-friendly interface were developed using HTML and CSS programming languages, and JavaScript was used to implement the embedded IGV interactive features on the website (Robinson et al. 2023). The website is currently being hosted on a UBC (University of British Columbia) server running Apache. We also set up a MySQL database management system to store the processed lion genome data on the same server and established a connection between the website and the database using PHP scripting language. The database schema was designed to include tables for storing all relevant information.

The website was designed to display all the data and results from our AA lion project, and the development process involved the use of a text editor and various web development tools. Manual testing was done for functionality and usability checks. The MySQL queries used to process and display the data were validated for accuracy and consistency. Some user feedback was also collected and incorporated into the development process, with adjustments made to the website based on this feedback. This website does not collect or process personal or sensitive data, and all ethical considerations were taken into account during its development.

## Supplementary Material

evae021_Supplementary_Data

## Data Availability

All variants are available in supplementary table S7, Supplementary Material online and at the accompanying webpage: http://chemogenomics.pharmacy.ubc.ca/lion-website/. All sequencing data associated with this study have been deposited in the GenBank Sequence Read Archive (SRA) as BioProject PRJNA1052129 and are available under the accession numbers SAMN38857731 to SAMN38857745.
