# Supplementary material for: The Addis Ababa Lions: Whole-Genome Sequencing of a Rare and Precious Population
Source: Genome Biol Evol. 2024 Feb 1;16(2):evae021. doi: 10.1093/gbe/evae021 (PMC10871700; doi:10.1093/gbe/evae021)
Supplement: evae021_Supplementary_Data [file evae021_supplementary_data.zip › Supp-figures-20231102.pdf]

Supplementary Figures

FigureS1. A) Verification of the AA genomes integrity: Amplification of the African lion-specific mitochondrial gene, *LIHY*, indicated one single band (206 bp) for all 15 individuals, B) Sex verification of the AA lions: All fifteen individuals indicated single bands (150 bp) on *KDM5C*. The eight males (colored in white) showed single bands (417 bp) on male-specific gene, *DDX3Y*, while the seven females (colored in blue) indicated non-specific binding for the same gene. The used ladder was 1 Kb Plus DNA ladder (Thermo Fisher, Cat# 10787026).

Figure S2. Number of individual lion variants observed when the AA genomes were aligned to the cat (felCat9), tiger (PanTig1.0), leopard (PanPar1.0) and African lion (PanLeo1.0) reference genomes. Fifteen variants label indicates variants detected in all fifteen AA lions. 2-14 variants are the ones found in 2-14 AA lions. Unique variants are those variants that were detected in only one AA lion when all AA lions were called for that specific locus.

Figure S3. Enriched gene expression pathways based on nonsynonymous SNPs and frameshift indels in AA lions when mapped to genomes of the domestic cat (felCat9, grey), tiger (PanTig1.0, red), leopard (PanPar1.0, yellow) and African lion (PanLeo1.0, blue) genomes. The nodes correspond to the number of genes associated with each pathway, and the edges connecting them illustrate the extent of shared genes between the nodes. The width of the edges is proportional to the number of genes that are common between the two connected nodes.

Figure S4. Plots of uncalibrated (before; in pink) vs recalibrated (after; in blue) bam files.

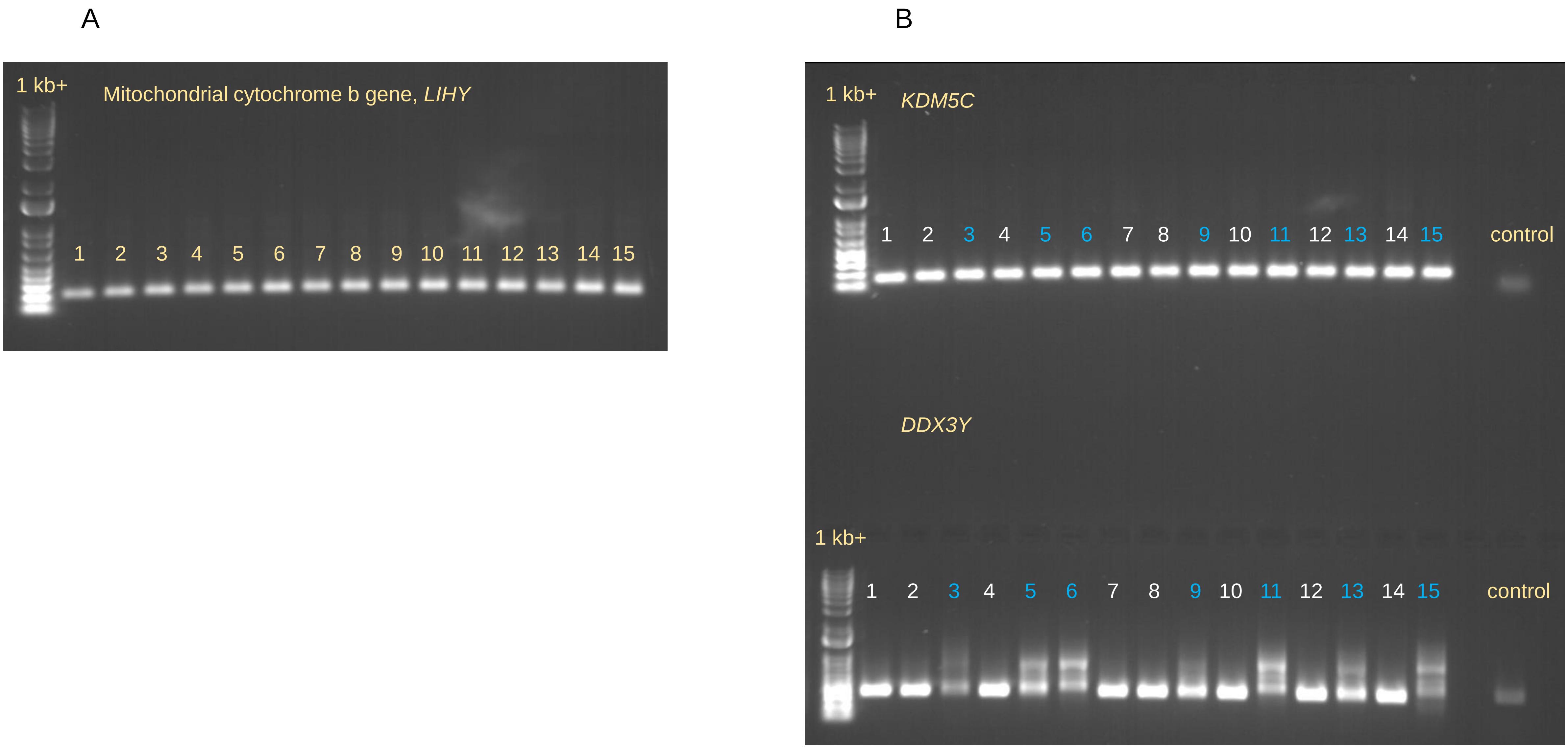
A
B
1 kb+
Mitochondrial cytochrome b gene, *LIHY*
1 2 3 4 5 6 7 8 9 10 11 12 13 14 15
1 kb+
KDM5C
1 2 3 4 5 6 7 8 9 10 11 12 13 14 15 control
DDX3Y
1 kb+
1 2 3 4 5 6 7 8 9 10 11 12 13 14 15 control

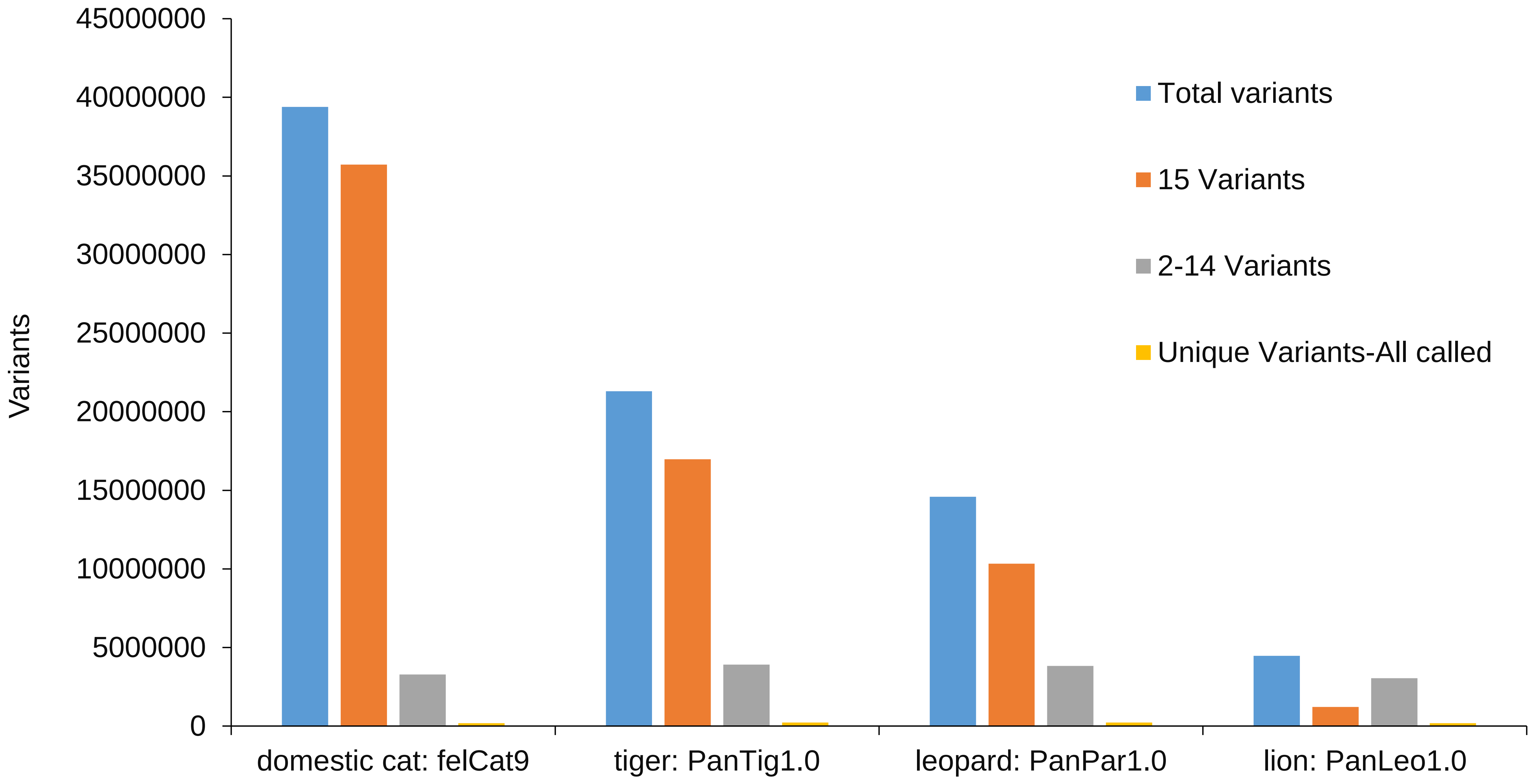
45000000
40000000
35000000
30000000
25000000
20000000
15000000
10000000
5000000
0
Variants
Total variants
15 Variants
2-14 Variants
Unique Variants-All called
domestic cat: felCat9
tiger: PanTig1.0
leopard: PanPar1.0
lion: PanLeo1.0

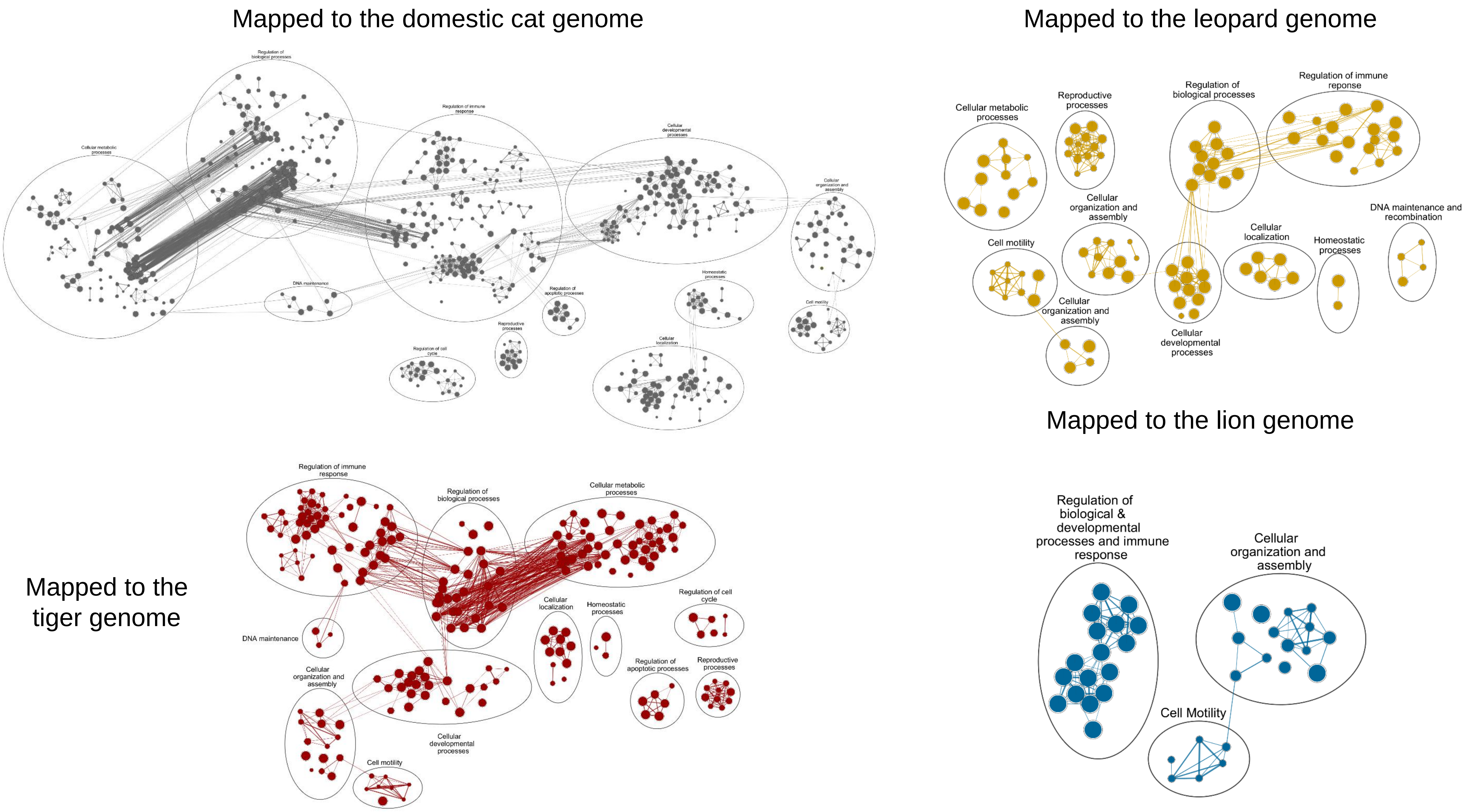

Mapped to the domestic cat genome
Regulation of biological processes
Regulation of immune response
Cellular developmental processes
Cellular metabolic processes
Cellular organization and assembly
DNA maintenance
Regulation of apoptotic processes
Homeostatic processes
Cell motility
Reproductive processes
Regulation of cell cycle
Cellular localization
Mapped to the leopard genome
Cellular metabolic processes
Reproductive processes
Regulation of biological processes
Regulation of immune reponse
Cellular organization and assembly
DNA maintenance and recombination
Cell motility
Cellular localization
Homeostatic processes
Cellular organization and assembly
Cellular developmental processes
Mapped to the lion genome
Regulation of biological & developmental processes and immune response
Cellular organization and assembly
Cell Motility
Mapped to the tiger genome
Regulation of immune response
Regulation of biological processes
Cellular metabolic processes
Cellular localization
Homeostatic processes
Regulation of cell cycle
DNA maintenance
Regulation of apoptotic processes
Reproductive processes
Cellular organization and assembly
Cellular developmental processes
Cell motility

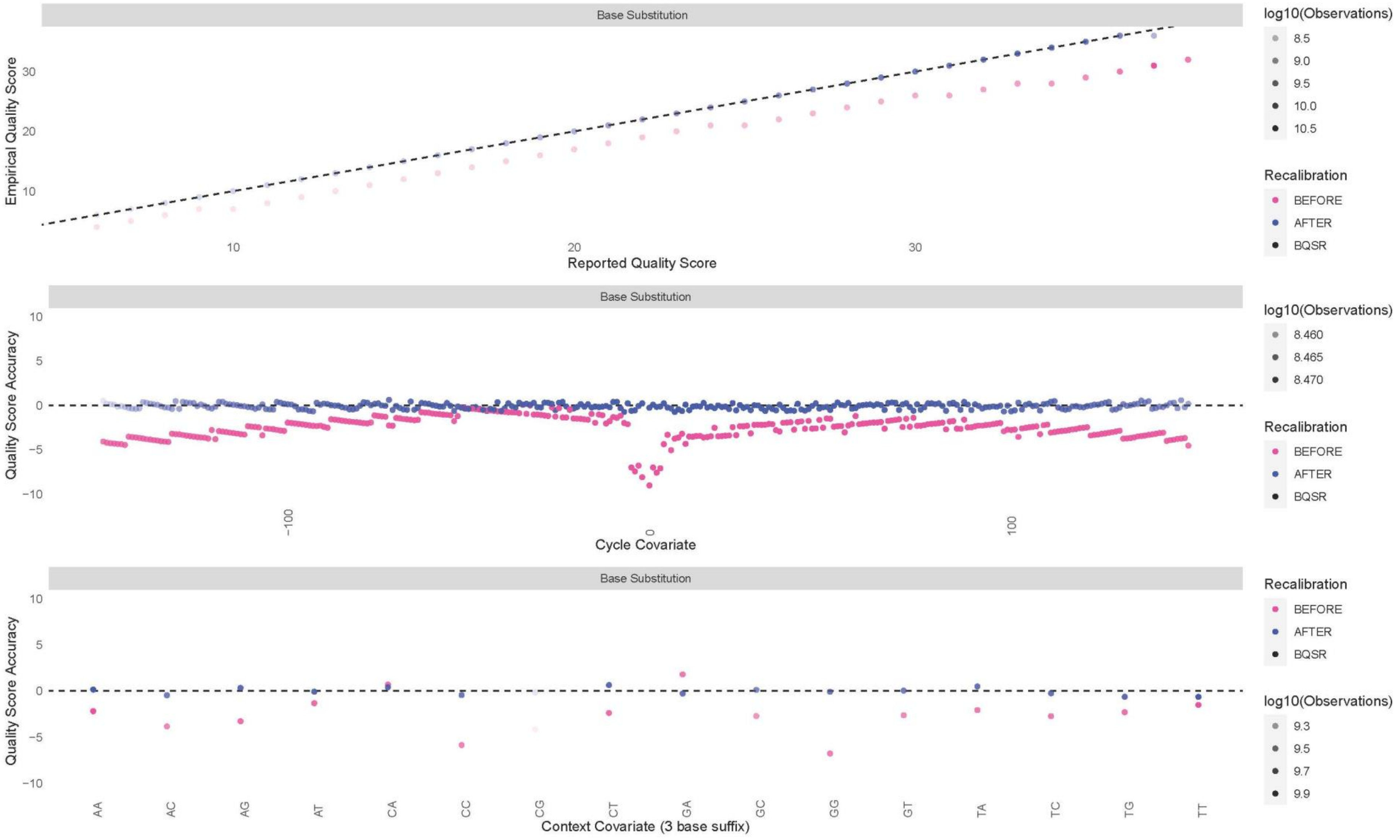

Base Substitution
Empirical Quality Score
Reported Quality Score
log10(Observations)
8.5
9.0
9.5
10.0
10.5
Recalibration
BEFORE
AFTER
BQSR
Base Substitution
Quality Score Accuracy
Cycle Covariate
log10(Observations)
8.460
8.465
8.470
Recalibration
BEFORE
AFTER
BQSR
Base Substitution
Quality Score Accuracy
Context Covariate (3 base suffix)
AA
AC
AG
AT
CA
CC
CG
CT
GA
GC
GG
GT
TA
TC
TG
TT
Recalibration
BEFORE
AFTER
BQSR
log10(Observations)
9.3
9.5
9.7
9.9
